# Incretin‐Based Adjunct to Background Insulin Treatment for Managing Body Weight Excess in Type 1 Diabetes: An Expert Opinion Viewpoint From the Italian Association of Clinical Endocrinologists

**DOI:** 10.1002/dmrr.70073

**Published:** 2025-09-07

**Authors:** Giuseppe Lisco, Anna De Tullio, Olga Eugenia Disoteo, Michela Armigliato, Edoardo Guastamacchia, Simonetta Marucci, Giovanni De Pergola, Enrico Papini, Marco Chianelli, Andrea Frasoldati, Vincenzo De Geronimo, Vincenzo Triggiani, The Italian Association of Clinical Endocrinologists—Medications and Therapeutics Committee; Diabetes Committee; Obesity and Eating Disorders Committee; Technology, Telehealth, and Digital Innovation Committee

**Affiliations:** ^1^ Interdisciplinary Department of Medicine School of Medicine University of Bari ‘Aldo Moro’ Bari Italy; ^2^ Division of Endocrinology, Diabetology and Clinical Nutrition Sant’ Anna Hospital—ASST Lariana Como Italy; ^3^ Chief of the Technology, Telehealth, and Digital Innovation Committee of the Italian Association of Clinical Endocrinologists Rome Italy; ^4^ Scienza dell'Alimentazione e Nutrizione Umana University Campus Biomedico Rome Italy; ^5^ Center of Nutrition for the Research and the Care of Obesity and Metabolic Diseases National Institute of Gastroenterology IRCCS ‘Saverio de Bellis’ Castellana Grotte Italy; ^6^ Unit of Endocrinology and Metabolism Regina Apostolorum Hospital Rome Italy; ^7^ Endocrinology Unit Azienda USL‐IRCCS di Reggio Emilia Reggio Emilia Italy; ^8^ Service of Endocrinology Morgagni CCD Catania Italy

**Keywords:** dual agonists, expert opinion, glucagon‐like peptide 1 receptor agonists, obesity, overweight, type 1 diabetes mellitus

## Abstract

Overweight and obesity represent common chronic metabolic disorders in the general population, and observed trends describe a substantial growth in the prevalence of weight excess also among individuals with type 1 diabetes (T1D), the so‐called ‘lean phenotype’ of diabetes. The sharp rise of weight excess and obesity‐related cardio‐nephron‐metabolic burdens observed in T2D is expected to produce similar consequences in T1D, leading to the urgent need to endorse therapeutic protocols as in most parts of the World no adjunctive treatments are approved for T1D, making weight excess management challenging in these individuals. The notable results shown by newer glucagon‐like peptide 1 receptor agonists (GLP‐1RAs) and emerging dual agonists, especially while managing cardio‐metabolic burdens, in T2D have encouraged fervent anecdotal and non‐anecdotal research also in T1D, indicating that non‐insulin injective agents can be effective and safe. With this expert opinion paper, the members of four of the most representative Committees of the Italian Association of Clinical Endocrinologists provide the scientific community with a state‐of‐the‐art report on the use of GLP‐1RAs and other incretin‐based injective therapy to treat body weight excess in T1D individuals and new insights on the topic with reliable methodology and clinical expertise.

**Abbreviations**
BMI: Body Mass Index
GIP: Glucose‐Dependent Insulinotropic Polypeptide
GLP‐1RAs: Glucagon‐Like Peptide 1 Receptor Agonists
HbA_1c_
: Glycated haemoglobin
RCTs: Randomized Controlled Trials
T1D: Type 1 Diabetes
T2D: Type 2 Diabetes

## 1 Background

Overweight and obesity represent common chronic metabolic disorders in the general population, with an estimated worldwide prevalence of approximately 40% [1]. Despite considerable variability among States due to social, economic, nutritional, and geographical factors, observed trends show a remarkable global increase in the prevalence of weight excess since childhood in both sexes [2].

Observed trends also reported a substantial increase in the prevalence of weight excess among individuals with type 1 diabetes (T1D), traditionally considered the ‘lean phenotype’ of diabetes [3]. Belgian epidemiological registry data show increasing trends in the prevalence of obesity, from 12.1% in 2001 to 21.7% in 2022, and metabolic‐related complications, especially among T1D patients with poor glucose control and those with a heavy burden of chronic complications [4]. Apart from common risk factors predisposing to weight gain and excess, such as unhealthy eating patterns, lack of physical exercise, or poor sleep quality and quantity, T1D individuals are also exposed to disease‐specific risk factors [5]. These include non‐physiological insulin replacement due to current therapeutic insulin‐delivery strategies, with consequent unavoidable insulin resistance, excessive carbohydrate intake to prevent or treat hypoglycemic episodes, frequent basal and meal insulin mismatch, predisposing to high glucose variability, corrective insulin boluses, sugar intake, or both. T1D patients also have a family history positive for type 2 diabetes (T2D), inheriting genes involved in insulin resistance and obesity. Because of their familial background, these patients are prone to excess weight as a consequence of insulin replacement therapy [6].

Weight excess increases the risk of cardiovascular diseases also in T1D [7], as similarly observed in T2D [8], and it contributes to the development of chronic diabetes‐related complications [9, 10]. Moreover, weight excess and weight gain in childhood are also associated with a 2‐fold higher risk of incident T1D in predisposed individuals. In contrast, significant weight loss (−10% compared to baseline) may prevent or delay T1D onset in around 20% of cases, suggesting relevant therapeutic implications of weight management in those individuals [11]. The reasons explaining the relationship between weight excess and beta‐cell failure in T1D are very similar to those described in T2D and include glucose‐ and lipo‐toxicity, insulin resistance, compensatory hyperinsulinemia, enhanced islet oxidative stress, and chronic low‐grade inflammation [12]. Augmented intestinal permeability and diet‐induced microbiota composition alterations are also associated with an increased risk of T1D in susceptible individuals [13].

Another issue includes the T1D endotypes. Five endotypes of insulinopenic autoimmune diabetes are recognized as specific entities due to genetic setting, immunological, demographic, and phenotypic (including body weight) characteristics. Endotype 1 is characterised by early onset and rapid progression of T‐ and B‐lymphocyte islet infiltration, high levels of circulating autoantibodies (especially insulin autoantibody), fast and remarkable insulin reserve decline, and low body weight. Compared to endotype 1, endotype 2 T1D is characterised by a slightly delayed onset, less rapid over time decline in serum c‐peptide levels and body weight, lower autoantibody titres with anti‐glutamic acid decarboxylase autoantibodies being predominant over anti‐insulin antibodies, and higher body weight (usually in the normal range). Endotypes 3, 4, and 5 identify three subsets of the latent autoimmune diabetes of adults classically recognized as a slow progressive form of autoimmune insulinopenic diabetes and identified by serum c‐peptide respectively < 0.3, 0.3–0.7, and > 0.7 mmol/L and phenotypically similar to T2D [14]. Continuous glucose monitoring provides useful information about beta‐cell reserve, probability of diabetes remission soon after the diagnosis, and correlation with body weight and insulin requirement, as demonstrated by Pollè et al. [15].

Despite the emerging topic, small sections of guidelines are specifically dedicated to weight management in T1D, and most emphasis is placed on insulin delivery strategies to attenuate weight gain, lifestyle correction, and some mentions of metabolic surgery for severe cases [16, 17, 18, 19]. The reason why recommendations are lacking is that evidence on non‐insulin pharmacotherapy in T1D is significantly sparser than in T2D [20, 21].

The present document is an expert opinion paper focussing on the historical progress registered with incretin‐based adjunctive treatment to prevent weight gain or treat weight excess and provides a viewpoint and new insights mostly on non‐insulin injective treatment in T1D.

## 2 Methods

PubMed/MEDLINE, the Cochrane Library, ClinicalTrial.gov, and Institutional and Scientific Society websites and documents were searched. Research words and Medical Subject Headings terms were used to conduct bibliographic searching using variable combinations (Supporting Information S1: research string and PICO). The research string of biomedical literature databases is shown in the Supporting Information S1. Database searching resulted in 81 results from 2005 to 2025, including case reports, randomized, and non‐randomized clinical trials. Data selection underwent further restriction from 2010 to 2025, resulting in 65 results discussed in the sections ‘The rationale for administering incretin‐based therapies in T1D’ and ‘Anecdotal experiences of the use of incretin‐based therapy in T1D’. Additional literature sources were from all coauthors, including references for the remaining sections.

The risk of bias of randomized clinical trials and the critical appraisal of randomized clinical trials and their certainty levels according to the GRADE method are reported in Supporting Information S1: Figure S1–S3.

## 3 The Rationale for Administering Incretin‐Based Therapies in T1D

Pivotal studies indicated that glucagon‐like peptide 1 receptor agonists (GLP‐1RAs) stimulate mice progenitor cells to proliferate and differentiate into insulin‐secreting cells [22], highlighting the therapeutic potential of these medications in T1D (i.e., adjuvant treatment in islet transplantation) [23]. Similar results were also reported for human embryonic and mesenchymal stem cells [24, 25]. Also, the results of a glucocorticoid‐induced glucose metabolism impairment model showed that GLP‐1RA administration prevented beta‐cell dysfunction and consequently hyperglycemia by stimulating insulin secretion and delaying gastric emptying [26]. Experimental data in humans also indicated that the addition of GLP‐1RAs to insulin in normal‐weight T1D patients with detectable beta‐cell reserve is associated with glucose control improvement, reduces insulin requirement, stimulates endogenous insulin secretion (as expressed by a 3.5‐fold increase in serum c‐peptide concentration) and autonomy from insulin therapy in 5 of 11 patients treated over a period of 12 weeks [27].

Background evidence suggests GLP‐1RAs could have a protective role on beta‐cells, stimulating insulin secretion, possibly downregulating pancreatic autoimmunity, and ameliorating glucose control in the early treatment of T1D and in patients with reduced pancreatic reserve.

By delaying gastric emptying, GLP‐1RAs also mitigate postprandial glycemic excursions and reduce food intake and insulin bolus requirement, eventually resulting in weight loss [28, 29, 30, 31]. Moreover, GLP‐1RAs suppress glucagon secretion in a glucose‐dependent manner and can play a relevant therapeutic role in counteracting alfa‐cell dysfunction classically observed in T1D [32].

### 3.1 Preliminary Evidence With Liraglutide in T1D: Effect on Body Weight and Glycaemic Control

Years after GLP‐1RA approval for T2D, off‐label pharmacological interventions were tested in patients with T1D and metabolic complaints. Liraglutide, in add‐on to background insulin treatment, was found to reduce fasting and daytime glucose levels and glucose variability [33]. Liraglutide also induced a slight improvement in glucose control, as expressed by glycated haemoglobin (HbA_1c_) levels, or reduced the mean daily insulin dose requirement regardless of residual beta‐cell function [34, 35]. The evidence also indicated that Liraglutide significantly decreased body weight compared to the multi‐daily insulin regimen alone.

Kuhadiya et al. characterised the ‘metabolic effect’ of Liraglutide in T1D obese individuals with clinical and laboratory signs of metabolic syndrome. Besides a slight but significant amelioration of glucose control and neutral effect on the hypoglycemic risk, patients achieved a significant weight loss (around 5 kg), reduced total and mealtime bolus insulin doses by 10 units/day, and improved systolic arterial pressure in 6 months of treatment [36]. Similar findings were reported by Harrison et al. in long‐standing T1D patients. When added to a multi‐daily insulin regimen, Liraglutide reduced body weight by around 4%, HbA_1c_ by 0.4%, and daily insulin doses by 19% without affecting the hypoglycemic risk versus placebo after 10 weeks [37].

In long‐lasting T1D normal‐weighted patients, Frandsen et al. found that a 12‐week Liraglutide trial was associated with relevant weight loss (−3 kg) and insulin requirement without any significant changes in glucose control and hypoglycemic risk. Moreover, T1D patients randomized to Liraglutide showed a relevant increase in resting heart rate and systolic arterial pressure decrease [38].

Positive preliminary results were also reported by another cohort of T1D patients with poor glucose control and weight excess while on insulin pumps. Liraglutide 1.8 mg/day, compared to placebo, slightly ameliorated glucose control, reduced total daily dose requirement by 8 units and provided a remarkable weight loss of around 6 kg [39].

A detailed study addressed the effect of GLP‐1RAs on food consumption and body composition. Particularly, Schmidt et al. found that Liraglutide 1.8 mg daily, compared to placebo, in add‐on to continuous subcutaneous insulin treatment for 26 weeks significantly reduced fat and lean mass by 4.6 and 2.5 kg, respectively, and the frequency of snack consumption and daily calorie resulting from simple carbohydrate intake by 27% in T1D patients with excess weight [40]. Other reports found that GLP‐1RAs can improve body composition by selectively reducing the amounts of visceral adipose tissue [41] and arterial pressure levels and stimulating lipid oxidation and thermogenesis while preserving lean body mass [42].

Slight but statistically and clinically significant results were also reported in a cohort of 144 patients, mostly treated with Semaglutide (64%) or Liraglutide (34%), up to 18 months of follow‐up, suggesting the durability of GLP‐1RA effectiveness in add‐on to background insulin treatment [43]. These results, in line with other findings, indicated that GLP‐1RAs were more effective in treating weight excess and related complications rather than improving glucose control in T1D [44] regardless of background insulin reserve. Exenatide, compared to Liraglutide, provided equivalent results [45].

Overall, these outcomes were achieved due to several effects of GLP‐1RAs to reduce appetite, increase satiety, delay gastric emptying, modulate thermogenesis, and sustain counter‐regulatory hormone response to hypoglycemia [46, 47, 48].

However, when used at the maximum daily dose in patients with considerable excess weight, Liraglutide increases the risk of gastrointestinal side effects, leading to possible discontinuation of treatment and potential concerns about overall safety [49]. One of these concerns is related to possible malpractice while managing T1D patients with GLP‐1RAs. Gastric emptying delay may reduce the frequency of food consumption due to nausea or emesis. Consequently, patients can miss frequent bolus administrations, reduce their total daily insulin exposure, and be at considerable risk of severe hyperglycemia, ketosis, and ultimately ketoacidosis [50].

### 3.2 Preliminary Evidence With Dulaglutide in T1D: Effect on Body Weight and Glycaemic Control

In patients with detectable beta‐cell reserve, unsatisfactory glucose control (HbA_1c_ 8.3%), excess weight, and intermediate diabetes duration (12 years), Dulaglutide 1.5 mg/week as an adjunct to continuous subcutaneous insulin injection led to remarkable weight loss (−5.6 kg) compared to placebo (−0.5 kg) due to a significant calorie restriction (−400 kCal/day), especially from lipid intake after 24 weeks of treatment. Notably, no improvement in glucose control was registered in the same study period [51].

Real‐life experience with Dulaglutide revealed that GLP‐1RAs can also be effective and safe in normal‐weighted patients with poor glucose control and negligible beta‐cell reserve [52]. This result was also confirmed by a post hoc analysis of the AWARD‐2, 4, and 5 trials, which confirmed the same Dulaglutide efficacy in autoimmune insulinopenic patients as in T2D [53].

Overall, first‐generation GLP‐1RAs, including short‐ and long‐acting daily agonists, provide little but clinically relevant HbA_1c_ reduction and considerable weight loss, up to 6 kg, in 24 weeks of treatment [54] irrespective of baseline beta‐cell reserve.

### 3.3 Preliminary Evidence With Semaglutide in T1D: Effect on Body Weight and Glycaemic Control

Real‐world data from a retrospective study conducted on long‐lasting T1D individuals, mean age of 44 years, and weight excess found that Semaglutide, compared to no adjunctive treatment, improved glucose control and reduced body weight by around 7 kg in 12 months [55]. Other authors confirmed the effectiveness of Semaglutide (0.25–2 mg) in inducing and maintaining significant weight loss (−5% compared to baseline) up to 9 months of treatment, but they reported uncertain glycaemic effects [56].

Better results came from a double‐blind, randomized, crossover trial in which T1D patients with weight excess (obese, 63%) were treated with Semaglutide up to 1 mg (or the maximum tolerated dose) and followed up to 12 weeks, comprising 2 months of titration in add‐on to a background insulin pump and 4 weeks of full‐dose Semaglutide plus an automated insulin delivery system to reduce biases. Overall, all participants showed a significant body weight reduction by 5% compared to baseline and a slight but significant amelioration of glucose control. The authors reported a correlation between weight loss and improvements in time in range and HbA_1c_ levels, even if patients with baseline undetectable rather than detectable c‐peptide levels lost weight equally (around 6.5 kg) despite slightly but suggestive worse glycaemic results [57].

Grassi et al. reported real‐life data from long‐term T1D patients with excess weight (body mass index or BMI of 30.9 kg/m^2^) with substantially adequate glucose control, as indicated by a mean baseline time in the range of 73%, while on sensor‐augmented pump therapy. These patients were started on Semaglutide 0.5 mg weekly and followed up to 6 months, showing a significant weight loss by 11% compared to baseline as the result of lower carbohydrate intake (baseline, 131 g/day vs. study completion, 112 g/day) and lower daily bolus insulin requirement (baseline, 0.28 IU/kg/day vs. study completion, 0.24 IU/kg/day) [58].

Data from a real‐life study in obese (mean BMI of 35.4 kg/m^2^) T1D individuals with moderate glucose control (HbA_1c_ 7.3%, time in range 74% and time below range 1%) while on automated insulin delivery demonstrate that adding a GLP‐1RA to background insulin treatment (Liraglutide or Semaglutide), compared to placebo, resulted in mean body weight reduction of 11% and a reduction in daily insulin requirement by 15 units after 6 months [59].

At the same, real‐world evidence on the protracted adjunctive use (≥ 1 year) of GLP‐1RAs in T1D patients with a mean age of 55 years, BMI of 34 kg/m^2^, and unsatisfactory glucose control (all had HbA1c levels > 7%; mean value: 8.5%) revealed a remarkable weight (−8.4% from baseline) and HbA_1c_ (−0.5% from baseline) decrease and insulin sensitivity amelioration, without increasing the risk of diabetic ketoacidosis, and new onset or progression of retinopathy in those individuals who achieved rapid improvement of glucose control [60].

### 3.4 Preliminary Evidence With the Dual Agonist Tirzepatide in T1D: Effect on Body Weight and Glycaemic Control

The recent development of chimeric GLP‐1 and glucose‐dependent insulinotropic polypeptide (GIP) dual agonists, such as Tirzepatide, has significantly improved the management of diabetes and related comorbidities [61]. Compared to GLP‐1RAs, Tirzepatide has demonstrated a notable effect in inducing weight loss at a magnitude sufficient to counteract the pathophysiological link between weight excess, impaired glucose metabolism, and cardiovascular risk [62, 63]. Compared to GLP‐1RAs, Tirzepatide is associated with more extensive weight loss when added to background insulin treatment in T2D [64].

Proof of concept evidence indicates that Tirzepatide has promising results in T1D. Akturk et al. examined the effect of Tirzepatide added to insulin treatment in individuals with class II obesity and found that the dual agonist was able to improve glucose control (HbA_1c_ −0.59%) and induce relevant weight loss (−10% of baseline body weight) after 8 months of treatment [65]. Similar results were reported by Karakus et al. in a small cohort of long‐term T1D patients with a mean baseline BMI of 39.6 kg/m^2^, HbA_1c_ 7%, and mean daily insulin dose of 64 units delivered by an automated system. Daily insulin requirement was dampened by around 30%, mostly because of a reduction in bolus rather than basal insulin infusion rate, and body weight was decreased by 10 kg, compared to baseline, over the first 2 months of treatment [66].

Snell‐Bergeon et al. retrospectively compared the glucometabolic effects of Semaglutide and Tirzepatide, compared to no treatment, in T1D patients suitable to receive off‐label adjunctive therapy for weight loss and glucose control improvement. The authors found that Semaglutide and Tirzepatide respectively reduced body weight by 9.1% and 21.4% and improved glucose control (HbA_1c_, −0.54% with Semaglutide; −0.68% with Tirzepatide) in 12 months [67]. Similar results were reported from another cohort with comparable background characteristics and treated with Tirzepatide added‐on to background insulin replacement up to 1 year of follow‐up [68].

## 4 Anecdotal Experiences of the Use of Incretin‐Based Therapy in T1D

A case series by Wong et al. reported effective and safe results with GLP‐1RAs and Pramlintide, an orally administered amylin inhibitor, in add‐on to background insulin treatment in 3 patients with T1D and concomitant comorbid obesity. A 32‐year man with obstructive sleep apnea syndrome achieved a significant weight loss of 21 kg (−16% of baseline body weight) in 10 months of Semaglutide 0.25–1 mg/week (titration completed in 2 months) and Pramlintide (15 mcg before breakfast, lunch, and dinner). In the second case, a 68‐year woman on insulin pump with diabetic retinopathy, coronary artery disease, hypertension, hypothyroidism, and depression/anxiety lost 12.8 kg (−14% of baseline body weight) 7 months after Dulaglutide 0.75–4.5 mg/week (titration completed in 5 months) and Pramlintide (60 mcg × 3/day). Finally, a 49‐year woman with hypertension, hypothyroidism, and depression lost 14.6 kg (−17.9% of baseline weight) over 6 months of treatment with Semaglutide (0.25–0.5 mg/weekly) and Pramlintide (15–30 mcg × 3/day). All patients reported tolerable adverse events (such as nausea or emesis) and a substantial reduction of total daily insulin dose with a neutral or slightly ameliorative effect on glucose control and hypoglycemic risk [69].

International literature reports a case of effective short‐term treatment with injectable Semaglutide 0.25–0.5 mg weekly in a 27‐year woman with class I obesity (BMI 30 kg/m^2^) and T1D, poor glucose control (HbA_1c_ 12.9%), suboptimal adherence to insulin prescription, and binge eating with overnight eating. After 4 weeks of treatment, she lost 5 kg in weight, also showing a significant increase in time spent in euglycemia (from 9% to 44%), a remarkable reduction in the number and duration of overnight hyperglycemic spikes, and objective improvement of binge eating behaviour and anxiety. Any improvements disappeared when she discontinued Semaglutide because of insufficient supply after 2 months of treatment and appeared again when she started to assume Semaglutide intermittently during the subsequent 8 months. Overall, this report indicates that Semaglutide could have positive effects on the frequency of food and caloric intake and, consequently, on glucose control in T1D patients with disordered eating and eating disorders [70].

Another report described the case of a 23‐year‐old woman with T1D on hybrid closed‐loop technology, suboptimal glucose control (HbA_1c_ 7.4%), and class II obesity (BMI of 38 kg/m^2^). The mean daily insulin dose was 82 units, while she was started on tirzepatide 2.5 mg/week and titrated by 2.5 mg every 4 weeks up to 7.5 mg/week. After 3 months, the patients reported a doubled time in glucose range, significantly lower glucose variability, and relevant amelioration of overall glucose control (HbA_1c_ 6.9%). Moreover, the authors recorded a considerable reduction in the mean daily insulin dose of 25 units and daily carbohydrate intake by 24%, resulting in a significant weight loss (3 kg) [71].

A 34‐year woman with long‐lasting T1D, a recent history of weight gain in the range of mild overweight (BMI 26.9 kg/m^2^), and glucose control deterioration (HbA_1c_ 8.3%) was intensified with Semaglutide up to 1 mg/week in add‐on to background basal‐bolus treatment and reported marked weight loss (−12 kg) and significant amelioration of body composition (drop in fat mass by 15% and visceral fat mass by 7%) along with reduction of insulin requirement, and amelioration of glucose control [72].

Comparable results were described by Raven et al. in a young woman with long‐lasting T1D and negligible beta‐cell reserve with marked overweight (BMI of 29.3 kg/m^2^) and poor glucose control who was intensified with Semaglutide (0.25–0.5 mg/week). The case report showed significant weight loss (−16 kg), amelioration of insulin sensitivity with consequent insulin requirement reduction of 36%, and improvement of overall glucose control in 6 months, highlighting an adjunctive therapeutic role of GLP‐1RAs also in a patient with undetectable c‐peptide levels [73].

The use of Semaglutide and Tirzepatide has also been reported in T1D youth patients, confirming the effectiveness of incretin‐based adjunctive treatment as previously described in adults. However, the rapid onset of restrictive eating disorders associated with rapid and drastic weight loss is a potentially concerning complication of GLP‐1RAs, requiring prompt dose‐escalation or treatment discontinuation and referral to dieticians and psychotherapeutics for specialist consultation [74, 75].

## 5 The Evidence‐Based Approach of Incretin‐Based Therapy in T1D

A few randomized controlled trials (RCTs) have been conducted and published, providing the most relevant evidence so far available (Table 1), beyond ongoing investigation (Table 2). In the ADJUNCT ONE treat‐to‐target randomized trial, around 1400 individuals with long‐lasting (undetecteble c‐peptide), uncontrolled T1D and weight excess were randomized to receive Liraglutide at three different doses (0.6, 1.2, and 1.8 mg once daily) or placebo in add‐on to background insulin treatment [77]. The most positive results were from Liraglutide at 1.2 and 1.8 mg/day, widely prescribed for T2D. For instance, Liraglutide 1.8 mg compared to placebo reduced HbA_1c_ levels by 0.2%, body weight by 5 kg, and total daily insulin dose by 9% after 52 weeks. Adverse, especially gastrointestinal, events were more common in patients on Liraglutide than placebo and drove a slight but significant increase in the discontinuation rate, commonly occurring between the 8th and 12th week after randomization. Hyperglycemic events were comparable between the 4 groups, but hyperglycemia associated with ketosis was significantly more frequent among patients on Liraglutide 1.8 mg compared with those on Liraglutide 1.2 mg and placebo. The same trend was observed for symptomatic hypoglycemic episodes.

**TABLE 1:** Summary of the most methodologically relevant studies assessing the efficacy and safety of GLP‐1RAs and dual agonists in T1D.

Characteristics	ADJUNCT ONE	ADJUNCT TWO	Gutierrez et al.
Study design	Double‐blind, treat‐to‐target trial	Placebo‐controlled, double‐blind, parallel‐group trial	Retrospective registry study
ClinicalTrial.gov	NCT01836523	NCT02098395	—
Identification number			
Population	T1D (*n* = 1391)	T1D (*n* = 835)	T1D (*n* = 51)
Intervention	Liraglutide (0.6–1.8 mg/d)	Liraglutide (0.6–1.8 mg/d)	Tirzepatide (12.5–15 mg/w, 35.3% 7.5–10 mg/w, 41.2% 2.5–5 mg/w, 23.5%)
Comparator	Placebo	Placebo	—
Randomization ratio	3:1	3:1	—
Background treatment	MDI and CSII	MDI and CSII	CSII and MDI
Follow‐up (weeks)	52	26	52
CGM	n.a.	n.a.	Yes
Mean age (years)	44	43	50
Diabetes duration (years)	21	21	24
Baseline HbA1c (%)	8.1	8	7.6
Baseline weight (kg)	86	84	111
Baseline BMI (kg/m^2^)	29.5	29	36.1
Baseline body composition	N.A.	N.A.	N.A.
Baseline daily insulin dose (IU)	MDI, 60	MDI, 59	68
	CSII, 50	CSII, 49	
Change in HbA_1c_ (%)	Liraglutide, −0.43 to −0.54 (0.6–1.8 mg) Placebo, −0.34	Liraglutide, −0.24 to −0.35 (0.6–1.8 mg) Placebo +0.01	−0.9
Change in insulin requirement (% from baseline)	Liraglutide, +4 to −5 (0.6–1.8 mg) Placebo, +4	Liraglutide, −5 to −10 (0.6–1.8 mg) Placebo, −3	−31.6
Change in body weight (kg)	Liraglutide, −1.3 to −4 (0.6–1.8 mg) Placebo, +0.9	Liraglutide, −2.5 to −5.1 (0.6–1.8 mg) Placebo, −0.2	−8.5
Gastrointestinal adverse events (events/PYE or %)	Liraglutide, 1.3 to 2.7 (0.6–1.8 mg) Placebo, 0.76	Liraglutide, 0.8 to 2 (0.6–1.8 mg) Placebo, 0.4	Any, 29.4 Most common = nausea, 13.7
Discontinuation rate (%)	Liraglutide, 4.9 to 14.7 (0.6–1.8 mg) Placebo, 3.4	Liraglutide, 5.7 to 16 (0.6–1.8 mg) Placebo, 1	5.9
Hypoglycemic episodes^a^ (events/PYE)	Liraglutide, 15.7 to 16.5 (0.6–1.8 mg) Placebo, 12.3	Liraglutide, 21.3 (1.2 mg) Placebo, 16.6	29.4
Hyperglycaemic episodes^a^ (events/PYE)	Liraglutide, 29.5 to 33.5 (0.6–1.8 mg) Placebo, 34.7	Liraglutide 40 to 44.8 (Higher rate with 1.2 mg) Placebo, 45.7	N.A.
Hyperglycaemic events with ketosis (% of patients)	Liraglutide, 6.3 to 11.2 (0.6–1.8 mg) Placebo, 6.9	Liraglutide, 6.2 to 8.3 (0.6–1.8 mg) Placebo, 4.4	N.A.
Adjudicate events of DKA (n)	Liraglutide, 8 Placebo, 0	Liraglutide, 1 Placebo, 0	0

Abbreviations: CSII, continuous subcutaneous insulin injection; DKA, diabetic ketoacidosis; GCM, continuous subcutaneous monitoring; IU, international units; MDI, multi‐daily insulin injections; N.A., not assessed or reported; PYE, patient‐year of exposure; T1D, type 1 diabetes.

^a^ Hypos were defined as glycaemia < 70 mg/dL; hyperglycemia should be considered as severe or potentially dangerous hyperglycemia (> 300 mg/dL).

**TABLE 2:** The most relevant ongoing investigation on the adjunctive use of incretin‐based therapies in T1D patients with weight excess and related cardiometabolic complaints.

Identification number	Molecule(s)	Topic	Sponsor	Status
NCT06630585	Tirzepatide	GIP/GLP‐1RA as adjunctive to automated insulin delivery in adults with type 1 diabetes	University of Bern	Not yet recruiting
NCT06180616	Tirzepatide	Tirzepatide for the treatment of concurrent type 1 diabetes and overweight or obesity	Royal North Shore Hospital	Not yet recruiting
—	Semaglutide	Reducing cardiometabolic risk with Semaglutide in type 1 diabetes [76]	—	—
NCT03899402	Semaglutide (and dapagliflozin)	Triple therapy in T1DM	State University of New York at Buffalo	Recruiting
NCT06057077	Semaglutide	Semaglutide GLP1 agonists with Degludec basal‐bolus insulin in early type 1 diabetes to basal bolus	Ministry of health Saudi Arabia	Not yet recruiting
NCT06762314	Semaglutide (and empagliflozin)	Efficacy and safety of Empagliflozin or Semaglutide in overweight/obese patients with type 1 diabetes	Tanta University	Not yet recruiting
NCT06411210	Semaglutide	Obesity complicating type 1 diabetes: GLP‐1 analogue anti‐obesity treatment	Yale University	Recruiting

The ADJUNCT TWO trial results confirmed the efficacy of Liraglutide, especially at 1.8 mg/day, when compared to placebo in improving glucose control and reducing body weight and insulin requirement [78]. From a safety viewpoint, the authors reported a significant increase in the risk of hypoglycemia only for Liraglutide 1.2 mg/day and hyperglycemia with ketosis only for Liraglutide 1.8 mg/day compared with placebo. Notably, patients with baseline c‐peptide levels above the lower value of the reference range achieved better glucose control and lower rate of hyperglycaemic event with ketosis, suggesting that background beta‐cell reserve is an important determinant of the clinical response to GLP‐1RAs from a glucocentric viewpoint [79].

A post‐hoc analysis of ADJUNCT ONE and ADJUNCT TWO trials, in fact, confirmed that patients with longer‐standing T1D and reduced beta‐cell reserve, as attested by undetectable c‐peptide levels, before starting GLP‐1RAs are more likely to discontinue the adjunctive treatment due to adverse effects or GLP‐1RA intolerance than individuals with residual insulin production. Also, a lower baseline BMI was associated with a higher probability of Liraglutide discontinuation [80].

Data from a retrospective registry study investigated the efficacy and safety of the dual GIP‐GLP‐1RA Tirzepatide as adjunctive treatment in T1D patients with overweight or obesity, most having class III obesity (mean BMI of 41.2%) [81]. The median study observation was 8 months, ranging from 3 to 12 months, and patients assuming Tirzepatide < 3 months were excluded. At the median observation time, body weight loss was significant (−8.5% compared to baseline), rising to −12.2% after 1 year of adjunctive treatment. The authors also reported a substantial reduction in HbA_1c_ levels by 0.9% and insulin requirement, both basal and bolus, by 31.6% during the first 6 months of treatment. Amelioration of glucose control reflected improvements in continuous glucose metrics (90% of patients wear a glucose sensor), such as a gain in time in the range from 51% (baseline) to 69% and a considerable reduction in time above the range from 48% to 29%. Other improvements included amelioration of arterial pressure profile, especially diastolic values, and a reduction of total and low‐density lipoprotein cholesterol and triglycerides. Nausea was the most common adverse event.

The results of a systematic review and meta‐analysis of RCTs, including 2389 patients, suggested that incretin‐based therapy, particularly GLP‐1RAs and not gliptins, was associated with a reduction of HbA_1c_ levels by 0.17% [−0.24; −0.11, *p* < 0.001], total daily insulin dose by 5.5 units/day [−8.89; −2.17, *p* = 0.001], and body weight by 3.24 kg [−4.43; −2.04, *p* < 0.001]. The hypoglycemic risk was neutral, while the studies found a remarkable increase in the risk of gastrointestinal side effects with frequency, onset time, and severity similar to those observed in T2D trials [82].

## 6 Real Need to Actions: An Expert Opinion

Around 1 in 3 T1D patients declare unmet needs by current treatments, including the use of technology, with the most difficulties for patients encountered in achieving optimal glucose control, disease management‐related fatigue, and weight control. Almost all responders were ready to use adjunctive treatment to effectively face their unmet needs, preferring oral rather than injective therapies [83].

The rationale of adjuvant treatment in T1D can be basically attributed to the effect of such therapy on supraphysiologic hyperinsulinemia generated by insulin replacement. Supraphysiologic iatrogenic hyperinsulinemia fosters weight gain, relevant glycaemic excursion, paradoxical increase of hypoglycemic risk, increased food intake, and further insulin requirement. The phenomenon is particularly evident in overweight and obese T1D individuals in whom adjuvant non‐insulin treatment may be essential to ameliorate insulin sensitivity, attenuate glycemic excursions, improve overall glucose control, reduce insulin requirement, lessen food and carbohydrate intake, and consequently induce weight loss or avoid weight gain [84].

**FIGURE 1:**
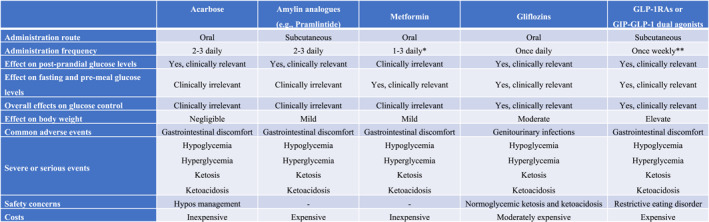
Qualitative appraisal of adjuvant non‐insulin therapy effectiveness and safety in T1D. *Slow‐release Metformin is an alternative and more expensive formulation than the rapid‐release one, and it can be administered once or twice daily. **First‐generation GLP‐1RAs are administered once daily; Semaglutide is registered as both oral and injectable formulations.

Nevertheless, adjuvant non‐insulin therapy use is off label in T1D, and there are no univocal or endorsed protocols. Several medications, whose mechanism of action leads to calorie loss, reduces appetite, or ameliorates insulin sensitivity, have been used for this specific purpose, including alpha‐glucosidase inhibitors, amylin analogues, biguanides, gliflozins, and GLP‐1RAs (Figure 1).

Acarbose, an inexpensive alpha‐glucosidase inhibitor agent, competes with disaccharides at the intestinal brush border of the small intestine, where packed microvilli express large amounts of alpha‐glucosidase responsible for the digestion of small carbohydrates. Due to this mechanism, Acarbose delays and reduces postprandial glucose absorption and consequently hampers postprandial glucose excursions and prandial insulin requirement [85]. As adjunctive therapy to insulin replacement in T1D, despite ameliorating postprandial glucose profile, Acarbose did not demonstrate to significantly reduce HbA_1c_ levels [86]. Moreover, although Acarbose does not increase the hypoglycemic risk, its adjunctive use in T1D may delay carbohydrate absorption, especially disaccharides, when treating a hypoglycemic episode orally [87]. Regardless of glucose control, Acarbose induces a slight and (probably) clinically unsatisfactory weight loss ranging from −1.3% to −2% from baseline in overweight and obese individuals, respectively [88].

Pramlintide, an injectable amylin analogue, binds to its specific receptors in the hindbrain and attenuates appetite, enhances satiety, decelerates gastric emptying, and suppresses glucagon secretion with the result of reducing food intake, mitigates glycemic excursion, and decreases insulin requirement, especially prandial insulin requirement [89]. The neurohormone peptide amylin is physiologically co‐secreted with insulin, and T1D patients have a double deficiency of insulin and amylin incretion. Therefore, Pramlintide can efficiently replace the hormone in T1D [90], improve glucose control, and moderately reduce body weight in a magnitude depending on baseline weight excess [91, 92]. Nonetheless, Pramlintide is approved as adjuvant therapy for T1D in the United States but not in Europe, where insulin replacement is the only treatment approved for insulinopenic diabetes [93]. The role of Pramlintide as adjuvant treatment in T1D is marginal due to its limited availability, frequency of injections (2–3 times a day), and costs [94].

Metformin, an inexpensive biguanide agent, usually induces slight glucose control and insulin sensitivity improvements [95] and modest weight loss or no weight effect in T1D [96, 97]. As observed with Acarbose, Metformin increased the risk of gastrointestinal adverse events and hypoglycemia [98].

In line with unmet needs and preferences, more recently, patients perceive real benefit from the adjunctive use of GLP‐1RAs and gliflozins to better control glucose levels, attenuate glycemic excursion, and obtain and maintain weight loss or avoid weight gain [99].

Gliflozins, compared to GLP‐1RAs (apart from oral Semaglutide), are administered orally, and this is the reason why T1D patients prefer them. Compared to Metformin, Gliflozins prompt more consistent improvement in glucose control (estimated mean difference in HbA_1c_ of 0.2%) and weight loss when added to insulin [100]. Real‐world data indicate that Gliflozins result in significant improvement of glucose control (HbA_1c_ −0.5%), weight reduction (−2.9 kg), and daily insulin requirement (−8.5%) by 12 months of adjunctive treatment [101]. Improvements were particularly evident in patients with background HbA_1c_ > 8% and BMI > 27 kg/m^2^ (−3.5 kg). These data align with other reports, indicating that combining Gliflozins with GLP‐1RAs may sustain more metabolic benefits than the single use of both classes of medications [102]. Dapagliflozin (5 mg) and Sotagliflozin (200 mg) were approved for use in T1D patients with weight excess and metabolic‐related complaints. However, the licence was withdrawn for both a few months after the temporary approval, probably because of safety concerns. Gliflozins considerably increase the risk of ketosis and ketoacidosis [103, 104, 105]. From a pathophysiological viewpoint, gliflozin‐related ketoacidosis differs from the classic form of diabetic ketoacidosis. Although both metabolic disorders are activated by insulin depletion, diabetic ketoacidosis results from an absolute and usually abrupt drop of insulin delivery, while gliflozin‐related ketoacidosis depends on a slight reduction of insulin requirement due to amelioration in glucose control and reactive hyperglucagonemia as a braking system to glycosuria and consequent calorie loss by urine [106, 107]. Compared to the rare frequency of diabetic ketoacidosis and severe hyperglycemia with ketosis episodes with other adjuvant agents, gliflozin‐related ketoacidosis is relatively more common, its onset subtle (usually named normoglycemic ketoacidosis) and requires more time to be treated [108]. On the other hand, Gliflozins have considerably higher costs than other oral agents.

More recently, a panelist expert opinion has recommended in favour of the adjunctive use of GLP‐1RAs to insulin pump (the most effective treatment in T1D) as emerging evidence supports the combination for ameliorating glucose control, reducing overall daily insulin requirement without increasing risks related to insulin depletion, and providing effective weight control or sustained weight loss [109].

As the last point, GLP‐1RAs (and gliflozins) have emerged over other non‐insulin agents as adjuvant therapy in T1D because of their cardio‐nephron‐metabolic protection properties [110, 111]. This is a non‐negligible viewpoint. T1D individuals are exposed to a high cardiovascular burden by age 20. Despite the young age of patients at the onset of diabetes, hyperglycemia, autoimmunity, and oxidative stress play a crucial role in driving the atherosclerotic background of possible future cardiovascular events. Years later, usually 15 to 20, hyperglycemia is complicated by other risk factors, such as hypertension, hypercholesterolemia, microalbuminuria, and excess weight that foster an additional increase in the probability of occurring cardiovascular events [112, 113]. Overall, there is evidence that cardiovascular events are uncommon before 35 years, but T1D patients are certainly exposed to a significantly higher life‐long cardiovascular risk than the general population and probably T2D patients.

Therefore, risk factor control and specific medications to reduce risks in primary prevention are desirable, including GLP‐1RAs [114, 115, 116, 117].

## 7 Expert Viewpoint and Conclusion

### 7.1 Expert Opinion Based on Ongoing Evidence

Careful attention should be given to weight excess in T1D because of its epidemiological trend and clinical implications on patients' health. Despite this topic, there is a relevant lack of specific evidence on the adjunctive incretin‐based treatment in T1D.

Data currently available are from pilot studies, anecdotal experiences, and a few well‐designed clinical trials that indicate considerable effects of GLP‐1RAs and dual agonists on weight loss, as observed in T2D [118]. The effect is mostly attributable to significant gastric emptying delay, positive satiety modulation, and negative appetite modulation, resulting in food intake and pharmacological‐induced caloric restriction [119].

Improvements in glucose control have also been described, and they seem more evident with injectable Semaglutide and Tirzepatide compared to older formulations. While the effects of adjunct incretin‐based therapy on overall glucose control, glycemic variability, risks of hypoglycemia, hyperglycemia, ketosis, and ketoacidosis largely depend on background beta‐cell reserve, T1D duration, background insulin treatment, and overall disease‐management capabilities, weight loss is a steadier result to be achieved and is not associated with insulin reserve. The magnitude of weight loss depends on specific GLP‐1RA effectiveness, the maximum tolerated dose assumed by patients, and background weight excess. A few studies have addressed the effect of incretin‐based adjunct therapy on body composition, indicating an overall favourable impact due to more sustained fat and visceral adipose masses than lean fat‐free mass reduction.

### 7.2 Safety Concern and Risk Mitigation

With respect to safety concerns, the critical appraisal of available evidence suggests that poor baseline glucose control and insulin reserve increase the risk of adverse events, especially hyperglycemia, ketosis, and gastrointestinal discomfort, leading to GLP‐1RA intolerance, frequent dose de‐escalation, or early discontinuation. All complications raise potential concerns on the overall patients' safety and, possibly, on the cost‐effectiveness of adjunctive therapy. Despite these being rare events while under expert management, severe hyperglycemia, ketosis, and ketoacidosis could be the result of an exaggerated drop in insulin requirement because of fear of hypoglycemia following an initial insulin sensitivity improvement, or in case of missing insulin bolus or basal administration due to gastrointestinal side effects (nausea or emesis) hindering food intake or predisposing patients to prolonged fasting. Appropriate management and patient education are essential to prevent or mitigate possible adverse events, which should be extensively explained with related underlying mechanisms, and informed written consent should also be obtained before starting the treatment. Patients experiencing such adverse events should also be managed cautiously, and it is advisable for them to receive a dietician referral and dietetic support to improve glucose control and reduce carbohydrate intake [120]. Anecdotal evidence suggests that GLP‐1RAs may correct eating patterns in T1D individuals with disordered eating or eating disorders, including binge and overnight eating. It is unclear if the effect results from a simple modulation of appetite and satiety or, instead, from the incretin‐mediated improvement of specific behaviours [121], hedonic eating, and food addiction [122]. In some cases, especially in young individuals, severely restrictive eating patterns can be triggered with the initiation of incretin‐based adjunctive therapy, highlighting the existence of predisposed individuals with a background substrate of eating disorders [123]. These individuals usually show drastic and exceptionally rapid weight loss while on GLP‐1RAs and require specific management, including dose de‐escalation, GLP‐1RA discontinuation, and psychotherapeutic and dietary support.

### 7.3 Conclusion

Overall, there is a real need for action from a therapeutic viewpoint. GLP‐1RA and dual agonist use as adjuncts to insulin treatment should be encouraged in T1D patients who require proper weight excess management to prevent or attenuate the burden of related comorbidities and complications. The most relevant limitation of adjunctive incretin‐based treatment in T1D is its safety. The second limitation in nations with public or prevalently public healthcare systems is that off‐label adjunct therapies cannot be reimbursed, leading to relevant implications about cardio‐metabolic protection, cardiovascular prevention, and overall quality of care. Therefore, this topic is expected to raise potential ethical concerns compared to the management of other chronic diseases, and public healthcare policies are called into action. Beyond benefits and risks, cost‐effective analyses are necessary to provide evidence supporting a more extensive use of incretin‐based treatment as adjunctive therapy in T1D [124].

## Author Contributions

G.L., conceptualization, writing – review and editing, writing – original draft, visualization, methodology, validation. A.D.T., writing – review and editing, writing – original draft, visualization, methodology, validation. O.E.D., writing – review and editing, visualization, validation. M.A., writing – review and editing, visualization. E.G., writing – review and editing, visualization, validation. S.M., writing – review and editing, visualization. G.D.P., writing – review and editing, visualization. E.P., visualization, validation. M.C., writing – review and editing, visualization, validation. A.F., visualization, validation. V.D.G., conceptualization, writing – review and editing, visualization, validation. V.T., visualization, validation, supervision.

## Conflicts of Interest

The authors declare no conflicts of interest.

## Peer Review

The peer review history for this article is available at https://www.webofscience.com/api/gateway/wos/peer-review/10.1002/dmrr.70073.

## Supporting information


Supporting Information S1

## Data Availability

Data sharing is not applicable to this article as no new data were created or analysed in this study.
