# Supplementary material for: Incretin‐Based Adjunct to Background Insulin Treatment for Managing Body Weight Excess in Type 1 Diabetes: An Expert Opinion Viewpoint From the Italian Association of Clinical Endocrinologists
Source: Diabetes Metab Res Rev. 2025 Sep 7;41(6):e70073. doi: 10.1002/dmrr.70073 (PMC12414530; doi:10.1002/dmrr.70073)

**Supplementary material**

**Research string for PubMed/MEDLINE and Cochrane Library**

“((((Type 1 diabetes) OR (T1D)) NOT ((type 2 diabetes) OR (T2D))) AND ((((glucagon like peptide 1 receptor agonist*) OR (GLP-1RA*)) OR ((glucagon like peptide 1 receptor agonist*) OR (GLP-1RA*))) OR (((((((((semaglutide) OR (dulaglutide)) OR (exenatide)) OR (liraglutide)) OR (lixisenatide)) OR (teduglutide)) OR (tirzepatide)) OR (cagrisema)) OR (retatrutide)))) AND (body weight)”

PICO

Population: Adults (i.e., equal to or more than 18 years old) with type 1 diabetes mellitus.

Interventions: any GLP-1RA (i.e., exenatide, lixisenatide, liraglutide, dulaglutide, albiglutide, semaglutide) or dual GIP-GLP-1 receptor co-agonists (e.g., tirzepatide).

Comparators: placebo, no adjunctive treatment (e.g., randomized, and non-randomized trials), or no comparison (e.g., case reports).

(Primary) Outcome: weight loss expressed as mean difference, standardized mean difference, or percentage of weight loss from baseline.

Risk of bias of randomized studies (ADJUNCT ONE AND ADJUNCT TWO TRIALS): Figure S1 and S2

Figure S1

Figure S2

Critical appraisal of randomized clinical trials and certainty levels following the GRADE method (ADJUNCT ONE AND ADJUNCT TWO TRIALS): Figure S3

Figure S3


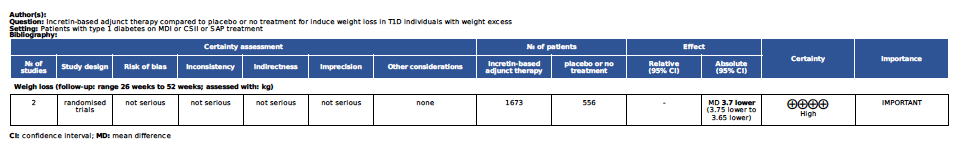

Supplement: Supplementary file 1 — Supporting Information S1 [file DMRR-41-e70073-s001.docx]
